# Detection of cardiac amyloidosis on routine bone scintigraphy: an important gatekeeper role for the nuclear medicine physician

**DOI:** 10.1007/s10554-024-03085-z

**Published:** 2024-03-23

**Authors:** Mohit Nebhwani, Karina Chaibekava, Anouk Achten, Marish I. F. J. Oerlemans, Michelle Michels, Peter van der Meer, Hans L. A. Nienhuis, Jerremy Weerts, Vanessa van Empel, Hans-Peter Brunner-La Rocca, Sandra Sanders-van Wijk, Jochem van der Pol, Christian Knackstedt

**Affiliations:** 1 Department of Cardiology and Cardiovascular Research Institute Maastricht (CARIM), Maastricht University Medical Center+, PO Box 5800, 6202 AZ Maastricht, The Netherlands; 2 Department of Radiology and Nuclear Medicine, Maastricht University Medical Center+, Maastricht, The Netherlands; 3 Department of Cardiology, Universitair Medisch Centrum, Utrecht, The Netherlands; 4 Department of Cardiology, Erasmus MC, Cardiovascular Institute, Thoraxcenter, Rotterdam, The Netherlands; 5 Department of Cardiology, University Medical Center, Groningen, The Netherlands; 6 Department of Rheumatology & Clinical Immunology, University Medical Center Groningen, Groningen, The Netherlands; 7 Department of Cardiology, Zuyderland Medical Center, Heerlen, The Netherlands

**Keywords:** Amyloidosis, Echocardiography, Bonescintigraphy, ^99m^Tc-HDP, Prevalence, Follow-up

## Abstract

Cardiac amyloidosis (CA)—mostly transthyretin-related (ATTR-CA)—has recently gained interest in cardiology. Bone scintigraphy (BS) is one of the main screening tools for ATTR-CA but also used for various other reasons. The objective was to evaluate whether all CA cases are detected and what happens during follow-up. All routine BS performed at the Maastricht University Medical Center (May 2012–August 2020) were screened for the presence of CA. Scans performed for suspected CA were excluded. A Perugini stage ≥1 was classified as positive necessitating further examination. The electronic medical record system was evaluated for any contact with cardiology or other specialists until 2021. Of the 2738 BS evaluated, 40 scans (1.46%; median age 73.5 [IQR: 65.8–79.5], 82.5% male) were positive (Perugini grade 1: 31/77.5%, grade 2: 6/15%, grade 3: 3/7.5%); the potential diagnosis ATTR-CA was not seen in 38 patients (95%) by the nuclear medicine specialist. During follow-up, 19 out of those 40 patients (47.5%) underwent cardiac evaluation without diagnosing CA. Available echocardiograms of patients with a positive BS showed left ventricular hypertrophy, a preserved ejection fraction, and diastolic dysfunction ≥2 in 9/47%, 10/53%, and 4/21% of patients, respectively. Additionally, 20 (50%) patients presented to at least one specialty with symptoms indicative of cardiac amyloidosis. The prevalence of a positive BS indicating potential CA in an unselected population is low but substantial. The majority was not detected which asks for better awareness for CA of all involved specialists to ensure appropriate treatment and follow-up.

**Graphical abstract:**

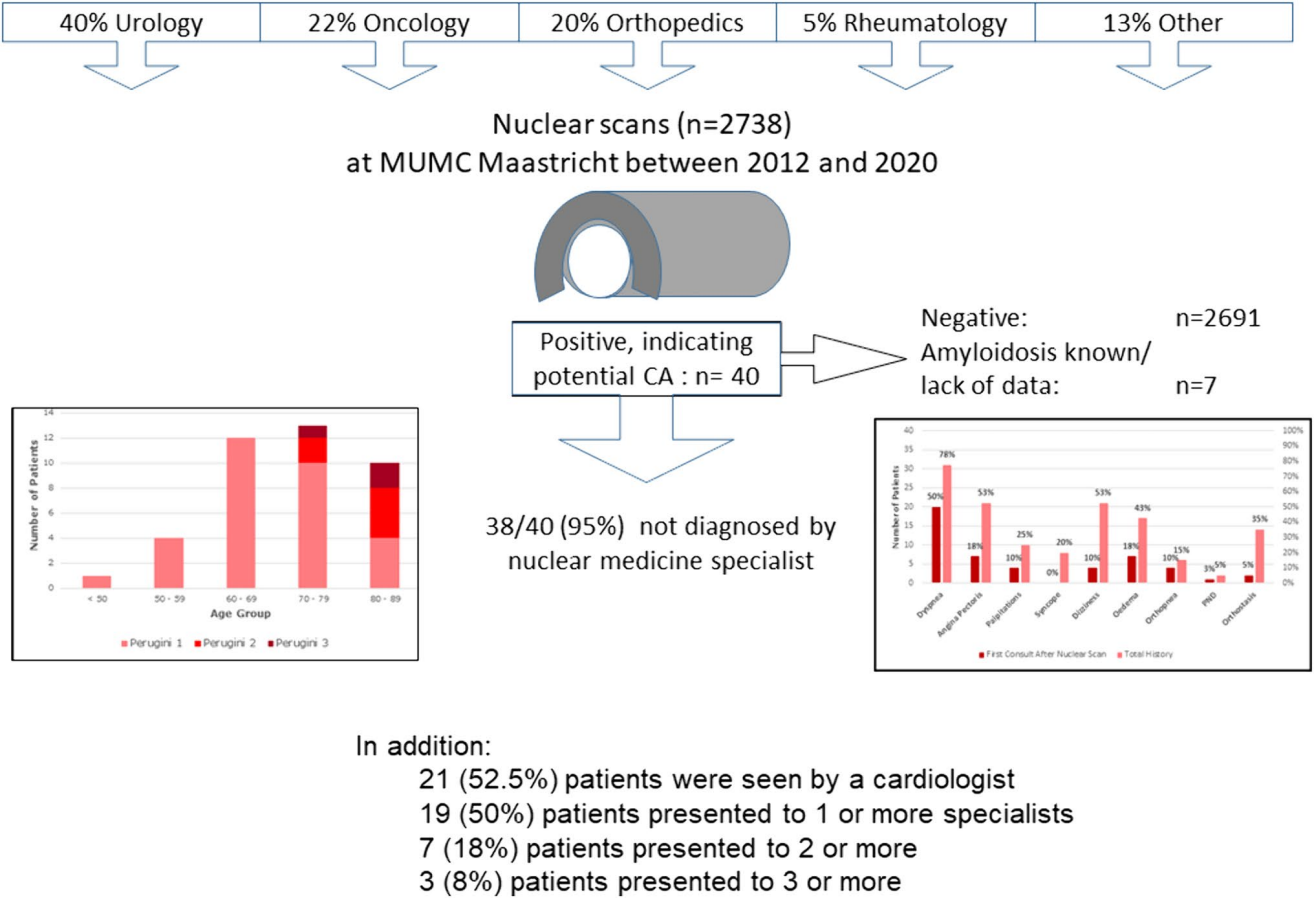

**Supplementary Information:**

The online version contains supplementary material available at 10.1007/s10554-024-03085-z.

## Introduction

Amyloidosis is a condition characterized by a deposition of misfolded proteins in multiple organ systems [1]. Probably the most clinically relevant form of cardiac amyloidosis (CA) is Transthyretin (TTR)-derived cardiac amyloidosis (ATTR-CA) inducing a deposition of misfolded TTR proteins [1, 2]. Cardiac involvement is the major determinant of mortality in this condition [3]. During recent years, medications have been developed for specific treatment of TTR that either halt production of or stabilize the TTR precursor protein, preventing further amyloid deposition in organs [2, 4, 5]. In addition, the prevalence seems to be higher than expected as ATTR-CA has been related to several cardiac conditions [6–10], yet diagnosis is often delayed or missed, delaying treatment and in hereditary cases, preventing appropriate familial screening.

Overall, early detection of cardiac involvement is crucial as existing organ depositions are not cleared by these drugs and potentially better treatment results are implied when installed early in the disease process [11, 12]. When the suspicion of CA is raised, there are distinct diagnostic pathways [13] defining the role of different modalities e.g. echocardiography but bone scintigraphy (BS) using different tracers has a prominent position [14–17]. On the other hand, BS has been used for decades for detection and analysis of a variety of benign as well as malignant osseous pathology [18]. Consequently, given the relatively high prevalence of ATTR-CA, particularly in the elderly population, incidental findings in up to 3.6% of all routinely performed BS been described [19–24]. A relevant portion of those incidentally identified patients already had signs of heart failure (HF) [20, 22, 23]. Also, the presence of ATTR-CA on BS was associated with increased mortality [22].

Still, there is only sparse information on the trajectory and follow-up of patients with a positive BS without prior suspicion of or known ATTR-CA. As improvement of awareness of the disease and optimization of hospital communication structures will potentially lead to better care of patients with ATTR-CA, insight into such processes is warranted. Therefore, this study aimed at describing characteristics and the diagnostic journey of patients whose BS were indicative of ATTR-CA.

## Methods

### Study population

**Fig. 1:**
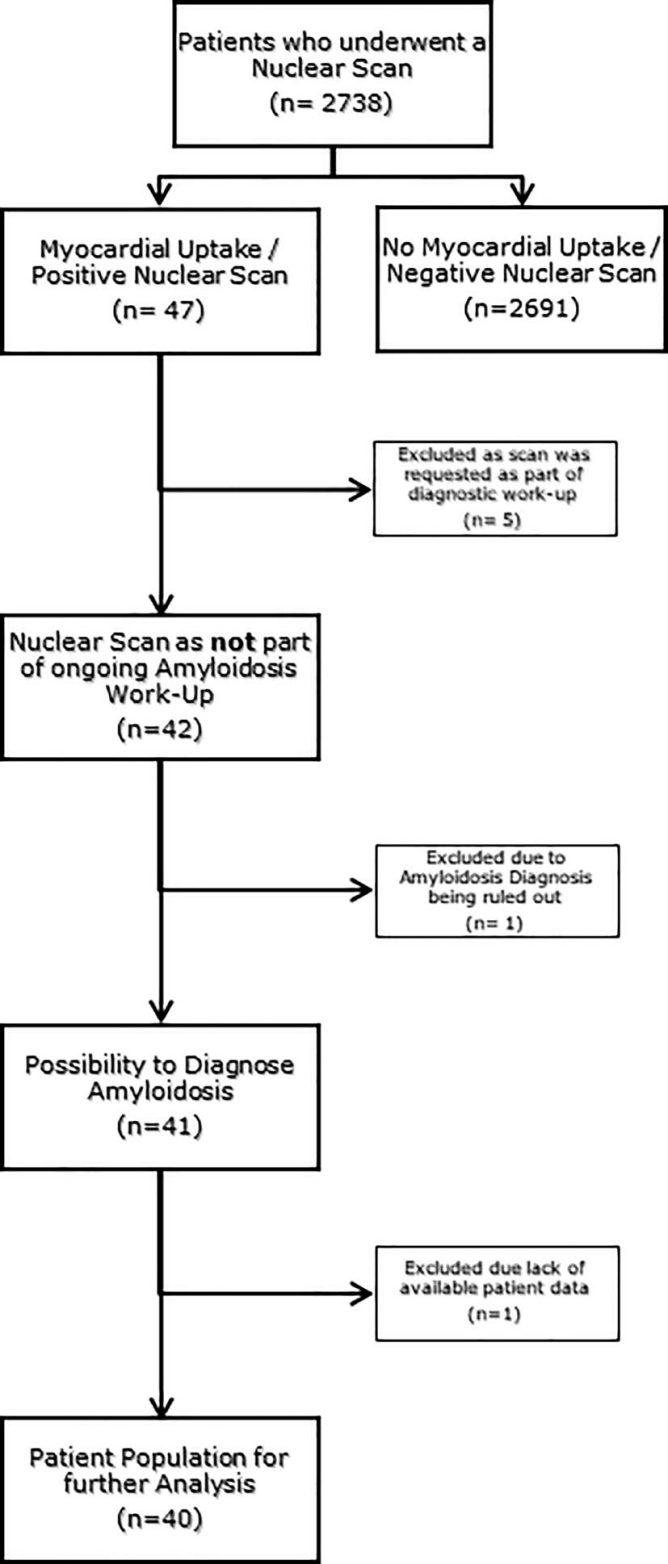
Flow diagram representing the inclusion and exclusion of patients leading to the patient population for further analysis

**Fig. 2:**
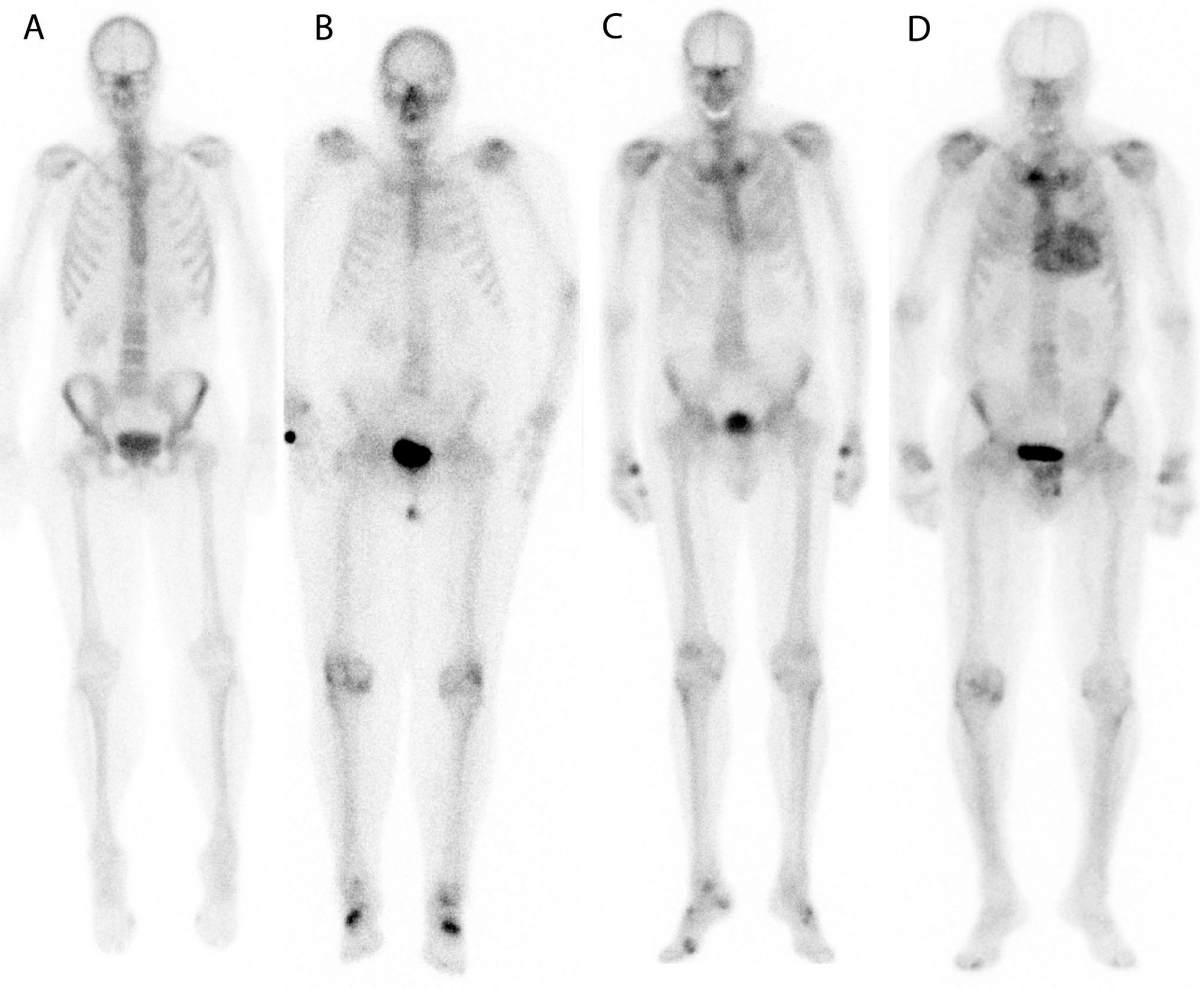
Depiction of different Perugini scores, **A **no tracer accumulation, **B** Perugini 1, **C** Perugini 2 and **D** Perugini 3

This retrospective, mono-centric, observational study, included all adult patients (n = 2738) who had undergone a BS depicting the thoracic region at the Department of Radiology and Nuclear Medicine at the Maastricht University Medical Center (MUMC+) in Maastricht, The Netherlands, between May 2012 and August 2020 (Fig. 1). All BS were performed at least 3 h after injection of Technetium-99m-hydroxy-methylene-diphosphonate (^99m^Tc-HDP). The BS was obtained according to the routine technique applied at the time it was performed. All scans were evaluated for myocardial tracer uptake by an evaluator blinded for clinical data (KC). Myocardial uptake was graded using the Perugini visual grading score (grade 0 = no myocardial uptake/normal bone uptake, grade 1 = cardiac uptake intensity less than bone, grade 2 = cardiac uptake similar or greater than bone, and grade 3 = cardiac uptake with much attenuated or absent bone signal; Fig. 2) [17]. All scans scored as Perugini grade ≥1 and all doubtable cases were reviewed by an experienced nuclear medicine specialist (JvdP). Scans with a Perugini grade ≥1 were considered as positive scans and were included in further data collection and analysis. There were no exclusion criteria. Also, BS that were performed as a screening for TTR-CA or to rule out TTR-CA were not included in the analysis. For patients with multiple scans, the first positive scan was taken as starting point and begin of follow-up. This project was approved by the Medical Ethics Review Committee (METC 2020-2429).

### Patient characteristics and amyloidosis work-up

Clinical data was collected from the electronic medical record system (SAP). This was performed by a second evaluator blinded to the results of the BS (MN). All relevant information (including medical history, hospital admission records, medication, diagnostic test data, and specialist outpatient reports) were obtained.

In addition, the reports of the nuclear specialist were thoroughly screened for the terms “myocardial uptake”, “amyloidosis”, “cardiac deposition” or any other description that could point to CA. Furthermore, it was evaluated whether the general description matched with the conclusion.

When evaluating the diagnostic work-up of amyloidosis, particular attention was paid to the clinical presentation of patients at their first outpatient cardiology contact and presence of additional information (magnetic resonance imaging, laboratory testing, endomyocardial biopsy, and/or genetic) *after the positive BS*.

Outcomes included hospital admissions due to cardiovascular problems and mortality. Elective procedures such as single-day admissions for electrophysiological interventions (pacemaker implantations, electro-cardioversions) were not included as events.

### Laboratory testing

Laboratory data for high sensitivity troponin T (hsTnT) and N-terminal pro-hormone of the brain natriuretic peptide (NT-proBNP) was automatically extracted from the electronic medical record system for patients before and after the positive BS. A cut-off value of 125 pg/ml was used for NT-proBNP [25] and a cut-off value of 14 ng/ml was used for hsTnT [26]. Additionally, patient records were screened for serum/urine immune fixation testing (M-protein) and determination of free light chain assays (serum/urine).

### Electrocardiography

Standard 12-lead electrocardiography (ECG) recordings were obtained when clinically available. Quantitative data (heart rate, PQ time and QRS duration) were automatically extracted from the electronic medical record system. Other, morphologic ECG abnormalities were scored manually: heart rhythm, low QRS voltage (defined as QRS amplitude ≤0.5 mV in all limb leads or ≤1 mV in all precordial leads), signs of left ventricular hypertrophy (conform Sokolow-Lyon-criteria and Cornell-criteria), pseudo-infarct pattern/pathological Q waves, R wave propagation and depolarization [11, 13].

### Echocardiography

Transthoracic echocardiography images (TTE) were obtained using Philips machines of different generations. Conventional parameters were evaluated according to recommendation [27] and verified at the central echocardiography laboratory at MUMC as a part of clinical care. The following cut-offs were used for determining cardiac pathology: LVEF < 50%, interventricular septum (IVST) or posterior wall thickness (PWT) > 12 mm, e′ lateral < 10 cm/s, e′ septal < 7 cm/s, E/e′ ratio > 14, TR-velocity ≥ 2.8 m/s, TAPSE < 17 mm, right ventricular (RV) S′ < 9.7 cm/s, LAVI > 34 ml/m^2^. Diastolic dysfunction was graded according to accepted algorithms, grade two or more was deemed pathological [28]. In addition, any changes indicative for CA e.g. aortic stenosis or pericardial effusion were registered.

### Statistical analysis

Characterization of the population was done using descriptive statistics. Categorical variables were presented using number frequencies and percentages whereas continuous variables using medians ± interquartile ranges (IQR) or mean ± standard deviation where applicable. Data was analyzed and graphically presented using the statistical program IBM SPSS statistics software version 25 (SPSS Inc, Chicago, IL).

## Results

### Patients

In the total cohort of 2738 patients who underwent a BS, a myocardial uptake (Perugini score ≥1) was observed in 40 patients (1.46%; 82.5% male, median age 73.5 [IQR: 65.8–79.5]); summarized in detail in Table 1. A Perugini grade 1 was observed in 31 (77.5%) patients, 6 (15%) patients showed a Perugini grade 2, and 3 (7.5%) patients were found to have a Perugini grade 3 (Supplement Fig. 1). There was a large variety of specialists ordering the BS (Supplement Fig. 2). The nuclear specialist mentioned cardiac changes pointing at TTR-CA in a total of merely 8 patients (20%). However, those findings were reported in the conclusion of the BS report in only 2 patients (5%), whereas the a potential TTR-CA diagnosis was missed in 38 (95%) patients.

**Table 1:** Baseline characteristics of the patient population

Baseline characteristics		
	(n = 40)	n%/[IQR]
Age at nuclear scan (years)	73.5	[65.8–79.5]
Sex (male)	33	82.5%
*Past medical history*		
Hypertension	29	72.5%
Diabetes	10	25.0%
Cerebral vascular event	12	30.0%
Atrium fibrillation/flutter	13	32.5%
Obstructive sleep apnea	4	10.0%
OSAS treatment	3	7.5%
*Interventions*		
Coronary artery disease		
PCI	5	12.5%
CABG	5	12.5%
Aortic valve		
Reconstruction	0	0.0%
Replacement	0	0.0%
TAVI	0	0.0%
Mitral valve		
Reconstruction	1	2.5%
Replacement	0	0.0%
Mitraclip	0	0.0%
Cardiac devices		
Pacemaker	3	7.5%
ICD	3	7.5%
*Renal function*		
GFR staging		
G1 or G2	13	32.5%
G3A	8	20.0%
G3B	9	22.5%
G4	8	20.0%
G5	2	5.0%
*Intoxications*		
Smoking status		
Current	4	*10.0%*
Former	20	*50.0%*
Median pack years	28.5	[18.1–42.5]
Heavy alcohol usage	2	*5,0%*

Data presented as n (%) or median [interquartile range (IQR)]

*OSAS *obstructive sleep apnea syndrome, *PCI* percutaneous coronary intervention, *CABG* coronary artery bypass graft, *TAVI* transcatheter aortic valve implantation, *ICD* implantable cardioverter defibrillator, *GFR* glomerular filtration rate

### Outpatient cardiology visits

**Fig. 3:**
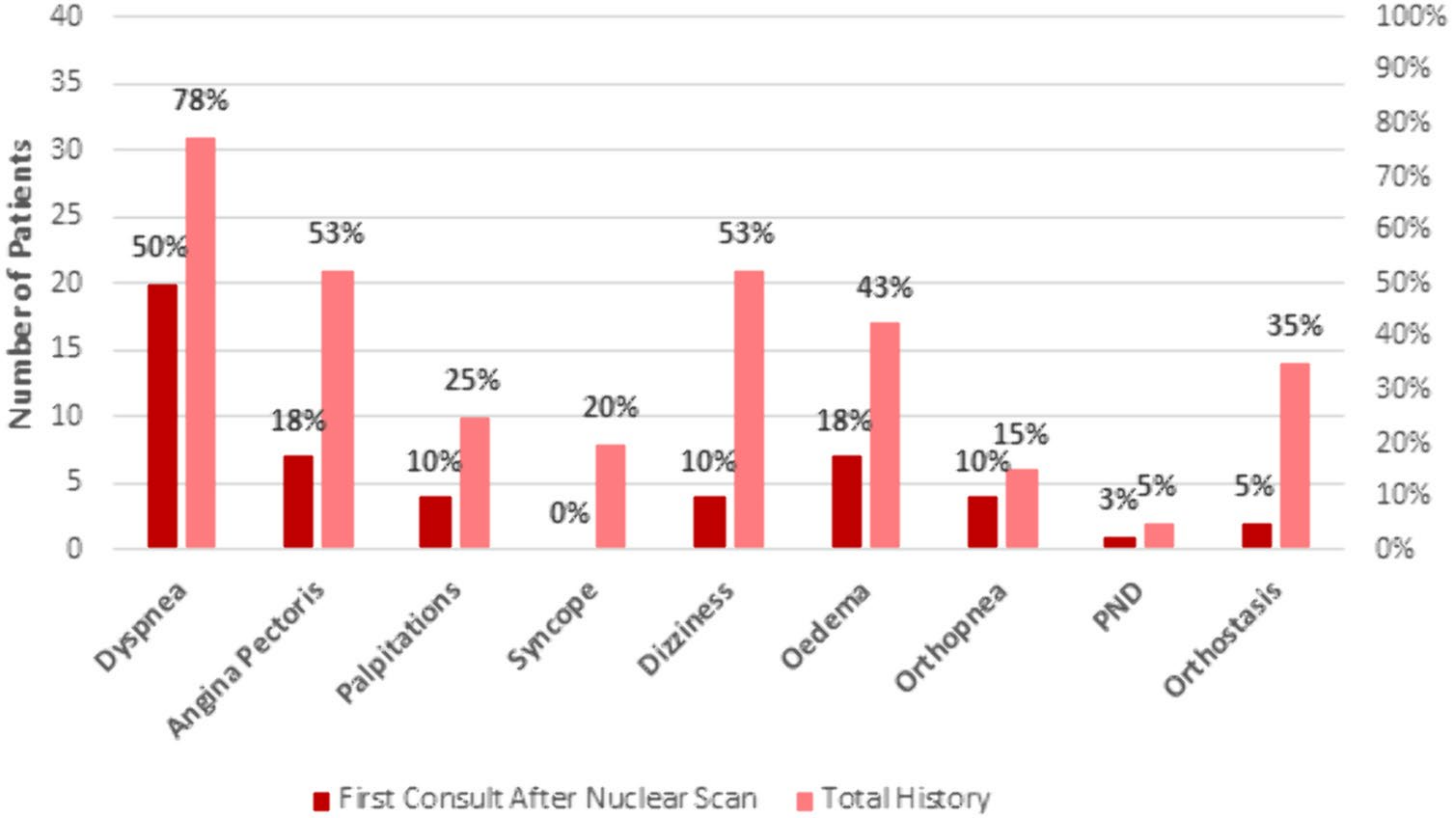
Cardiovascular symptoms: number of patients presenting with symptoms at their first consult after a positive scan vs. symptoms in their total history (*PND* paroxysmal nocturnal dyspnea)

Following the BS, 21 patients (52.5%) were seen by a cardiologist in the MUMC+ , details are provided in Table 2. The median timespan between BS and cardiology outpatient consult was 238 [115–933] days. The majority of patients presented with cardiac symptoms; details are depicted in Fig. 3. Nevertheless, the term “amyloidosis” was only mentioned in the outpatient cardiology report of two patients in the missed diagnosis group without further work-up or treatment. For the two patients correctly diagnosed by the nuclear medicine physician, it still took 241 and 1237 days for the cardiologist to consider CA diagnosis.

**Table 2:** Diagnostic work-up post positive nuclear scan

	Missed (n = 38)	Diagnosed (n = 2)	Total (n = 40)
*Cardiology consult*	19 (50.0%)	2 (100.0%)	21 (52.5%)
Amyloidosis symptoms present	10 (26.3%)	2 (100.0%)	12 (30.0%)
Amyloidosis mentioned in report	2 (5.3%)	2 (100.0%)	4 (10.0%)
Time to consult (days)	185 [106–881]	699	238 [115–933]
*CMR performed*	3	0	3 (7.5%)
Amyloidosis observed	0	–	0
Time to CMR (days)	1021 [232–1079]	–	1021 [232–1079]
*Myocardial tissue biopsy*	0	0	0
*Genetic testing*	3 (7.9%)	1 (50.0%)	4 (10.0%)
Variants for amyloidosis tested	0	1 (50.0%)	1 (2.5%)
Variant detected	0	0	0
Time to genetic test (days)	246 [157–923]	570	408 [201–827]
*M-Protein laboratory testing*			
Serum protein electrophoresis	13 (34.2%)	1 (50%)	14 (35.0%)
Urine protein electrophoresis	0	0	0
Serum FLC assay	5 (13.2%)	2 (100%)	7 (17.5%)
Urine FLC assay	0	2 (100%)	2 (5%)

Data presented as n (%) or median [interquartile range]

*CMR* cardiac magnetic resonance imaging, *FLC* free light chain

In addition, patients were seen by a variety of different specialists. The diagnostic work-up of patients in our study is summarized in Table 2 (cardiac) and Table 3 (non-cardiac). More specifically, they presented with a number of different findings suggestive of amyloidosis (Table 4). Overall, within the missed diagnosis group, it was observed that 19 patients (50%) presented to one or more specialists with amyloidosis symptoms, 7 (18%) patients presented to two or more, 3 patients (8%) presented to three or more, and lastly 1 patient (3%) presented to four or more specialists with symptoms indicative of amyloidosis.

**Table 3:** Outpatient consults per specialist in the missed diagnosis group including median number of consults and different doctors per specialist plus (IQR)

Outpatient specialist	Number of patients with an outpatient consult (n = 38)		Number of patients with amyloidosis symptoms		Median number of outpatient contacts per patient (IQR)		Median number of different doctors per specialist	
Neurologist	5	(13%)	2	(40%)	2	(2–4)	2	(1–3)
Dermatologist	12	(32%)	2	(17%)	1	(1–2)	1	(1–2)
Internist	24	(63%)	6	(25%)	1	(1–3)	1	(1–2)
Urologist	19	(50%)	2	(11%)	1	(1–3)	1	(1–2)
Geriatric Specialist^a^	2	(5%)	0	(0%)	4	(n/a)	2	(n/a)
Pulmonologist	6	(16%)	3	(50%)	1	(1–1)	1	(1–1)
Otorhinologist	8	(21%)	2	(25%)	1	(1–2)	1	(1–2)
Orthopedic surgeon	8	(21%)	2	(25%)	2	(1–3)	2	(1–3)
General surgeon	9	(24%)	3	(33%)	1	(1–2)	1	(1–2)
Gastroenterologist	6	(16%)	1	(17%)	1	(1–4)	1	(1–1)
Oncologist	14	(37%)	2	(14%)	1	(1–3)	1	(1–2)
Ophthalmologist	9	(24%)	2	(22%)	1	(1–3)	1	(1–2)

^a^No IQRs were included for the Geriatric Specialist due to the limited number of patients

**Table 4:** Non cardiac findings suggestive for amyloidosis

Extra-cardiac manifestations		
	(n = 40)	%
Carpal tunnel syndrome	4	10.0
Bilateral	4	10.0
Unilateral	0	0.0
Polyneuropathy	11	27.5
Spinal surgery	11	27.5
Dysautonomia	16	40.0
Ruptured biceps tendon	0	0.0
Lumbar spinal stenosis	6	15.0
Hearing loss	11	27.5
Bilateral hearing loss	7	17.5
Unilateral hearing loss	3	7.5
Sudden deafness	1	2.5
Cutis Laxa	0	0.0
Macroglossia	1	2.5
Skin discoloration	8	20.0
Skin bruising	3	7.5
Corneal lattice dystrophy	0	0.0
Vitreous deposits	3	7.5

### Laboratory testing

HsTnT and NT-proBNP were only determined in a small group of patients with a positive BS (12 (30%) and 16 (40%) respectively). When available, results were abnormal in >90% pointing to cardiac disease. In addition, serum immune-fixation electrophoresis and serum free light chain assay were provided in Table 2. Overall, a monoclonal protein peak in the gamma region of the protein spectrum was observed in only one patient. Serum free light chain assay in this patient was positive for an increase in kappa light chains.

### Echocardiography

In a relevant proportion of patients, TTE was performed prior to the BS (18; 45%). In the majority, there were changes potentially indicative for CA such as left atrial dilatation (16; 89%) or left ventricular hypertrophy (7; 39%) (supplement table 2a, b). TTE findings indicative for CA preceded BS with a median time of 1313 [IQR: 860–2036] days. On TTE performed after BS similar findings were seen (Supplement Table 3a, b).

### Electrocardiography

ECG was performed in 35 patients preceding positive BS, of whom 24 (68.6%) showed abnormalities suggestive for cardiac disease. ECG abnormalities presented several years before positive BS (median 4292 days; IQR [1–5787]). The most common ECG abnormalities were delayed intraventricular conduction (QRS ≥100 ms; 15; 45.7%), delayed R wave propagation (8; 25.7%) and an abnormal heart axis (8; 22.9%) (Supplement Table 4a). Also, after the scan, the majority of ECG were suggestive for cardiac disease in 88.2% (Supplement Table 4b).

### Other diagnostic examinations

Only a minority (6; 15%) of patients underwent cardiac magnetic resonance imaging, endomyocardial biopsy or genetic testing. Details are provided in Table 2.

### Outcomes

The median duration of follow-up was 1314 day [IQR: 699–2523]. There was a relevant number of cardiovascular-related hospital admissions (8, 20%). Also, mortality was high occurring in 24 (60%) patients. The cause of mortality was undetermined in 9 patients due to unavailable hospital record information. The follow-up from BS to death was 892 days [397–1353].

## Discussion

This study thoroughly evaluated the clinical trajectory of patients with an incidental finding of potentially indicative of TTR-CA. We showed that a positive BS was missed by the majority of the involved specialists: firstly, there was no explicit mention of CA in the report of the nuclear specialist, secondly, there was no follow-up on suggestive cardiac findings and, thirdly, other specialists did not draw correct conclusions based on non-cardiac findings either.

Overall, the prevalence of positive BS was low and comparable to other studies [19–23]. Of course, this mostly depends on which stages of Perugini are included (1–3 vs. 2–3). As we included all potentially positive scans including Perugini 1, we saw a slightly higher prevalence compared to Bianco et al. [20] or even Longhi et al. [23]. However, this prevalence was lower in comparison to other studies [22]. Still, a prevalence of 1.5% in the general population above 65 years would result in a significant number of patients with TTR-CA.

Probably due to the nature of the disease, an early and fast diagnosis is a challenge. Fragmentation of the care for these patients, heterogeneous presentation and non-specific symptoms—also because of other comorbidities—lead to late recognition [29]. This need for better awareness is nicely underlined by the fact that the majority of our population had one or even more appointments with numerous specialists. This is unfortunate as early treatment with new medication could allow to prevent progressive deposition of fibrils [4, 5]. However, despite the presence of cardiac symptoms and, other, non-cardiac “red-flags” [29], the potential diagnosis was missed in almost all patients. In addition, there were some clues as cardiac markers such as troponin T or BNP were elevated in most of the patients. However, echocardiography and ECG showed abnormalities but not in all patients [23]. Given the fact that patients were elderly with relevant co-morbidities, interpretation of those findings is challenging. Overall, there were several hints besides the presence of a positive BS which could have led to the correct diagnosis. However, cardiologist are known to underperform regarding the detection of CA [30].

Greater awareness for cardiac amyloidosis by the nuclear medicine physician is important. In this study, only the minority of positive scans was noted and only a few clearly mentioned in the conclusion of the report. This can be attributed partially to relatively low occurrence of cardiac tracer accumulation in a general population on one hand, as illustrated by this and other studies. On the other hand, the use of BS is aimed at diseases other than cardiac pathology in the general population, and consequently, nuclear medicine physicians have to be stringently focused on evaluation of the heart while analyzing the BS for a different purpose. Relatively high rates of Perugini grade 1 versus grade 2 or even grade 3 also provide a clue to the high fraction of missed diagnosis. Still, Perugini grade 1 clearly is an indication for further investigation. Furthermore, especially in an ageing population, limited cardiac accumulation can be obscured by costal cartilaginous uptake [31]. There might be a role of artificial intelligence to improve detection [32].

As many BS were ordered by colleagues probably not that familiar with CA e.g. orthopedic surgeons or urologist, information on a positive BS needs to be clearly stated in the report [20]. Still, this might not be enough and the potential clinical consequences should be mentioned as well as a quick referral is warranted to a department with adequate knowledge or even expertise center. Also, additional reporting of relative tracer retention would be desirable [33]. This might also hold true for the differentiation between “blood-pooling” and myocardial accumulation, although blood-pool versus cardiac accumulation can be differentiated by SPECT-CT [31]. Of course, there might be a learning curve within nuclear medicine as this topic has attracted attention in many specialties.

Eventually, there was a substantial mortality and hospitalization rate in the patients with a positive BS which is in line with other studies showing a correlation of outcome with the Perugini grade [20–22]. Still, those findings might differ due to the characteristics of the evaluated populations, specifically referring to the oncological background of patients and the presence of osseous metastases.

## Limitation

As this a retrospective study, we do not have a cardiac biopsy as gold standard in the patients. Therefore, we might overestimate the presence of CA given the possibility of a false-positive scan with a Perugini score grade 1. This also holds true for the differentiation between blood-pool versus cardiac accumulation of the tracer which was impossible in the majority of patients due to the absence of SPECT/CT. In addition, this score does not necessarily means cardiac amyloidosis and can also mean AL amyloidosis. Thus, Perugini grade 1 also had consequences as adequate diagnostic steps needed to be initiated. Also, there was no definite diagnosis on cardiac biopsy in all patients as a gold standard. However, the main aspect of this study was to depict the presence of any potential implication of CA. Also, the exact indication for the bone scan was not evaluated which could have influenced survival of patients.

In addition, there was a relevant amount of missing information e.g. serum/urine protein electrophoresis. Unfortunately, the local ethics committee did not grant us permission to contact individuals with a positive BS. Therefore, we were not able to perform additional examinations. Still, this focus of this study was the question what was missed.

Some patients might have been additionally examined at other centers. Consequently, the correct diagnosis might have been made earlier. However, MUMC serves not only as academic hospital but also as the first-line hospital in the region which is an unique situation within Limburg/The Netherlands. Therefore, almost all patients return to our center. In addition, any diagnosis in other hospitals should be mentioned in our hospital report system.

## Conclusions

There is a low but relevant prevalence of positive BS in an unselected population. The majority of those cases were not detected by most of the involved specialists. As those BS were not ordered by cardiologists, the role of the nuclear medicine physician is important. In addition, given the nature of CA, any specialist has to pay more attention to suggestive findings.

### Supplementary Information

Below is the link to the electronic supplementary material.

Supplementary file1 (PNG 9 KB) Age of patients with a positive nuclear scan per age group per Perugini Score

Supplementary file2 (PNG 12 KB) Inquiring specialist of the nuclear scan

Supplementary file3 (DOCX 17 KB)

Supplementary file4 (DOCX 19 KB)

Supplementary file5 (DOCX 19 KB)

Supplementary file6 (DOCX 21 KB)

## Abbreviations

ATTR-CA: Hereditary transthyretin-derived cardiac amyloidosis
BS: Bone scintigraphy
CA: Cardiac amyloidosis
EF: Ejection fraction
HF: Heart failure
hsTnT: High sensitivity troponin T
LV: Left ventricular
NT-proBNP: N-terminal prohormone of the brain natriuretic peptide
^99m^Tc-HDP: Technetium-99m-hydroxy-methylene-diphosphonate
TTR: Transthyretin
