# Supplementary figures and images for: Detection of cardiac amyloidosis on routine bone scintigraphy: an important gatekeeper role for the nuclear medicine physician
Source: Int J Cardiovasc Imaging. 2024 Mar 23;40(6):1183–92. doi: 10.1007/s10554-024-03085-z (PMC11213735; doi:10.1007/s10554-024-03085-z)

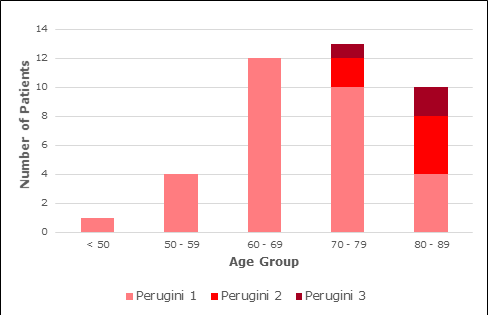

Supplement: Supplementary file 1 — Supplementary file1 (PNG 9 KB) Age of patients with a positive nuclear scan per age group per Perugini Score [file 10554_2024_3085_MOESM1_ESM.png]

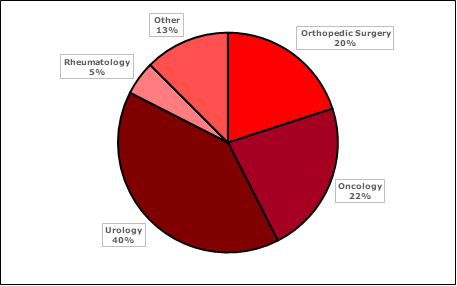

Supplement: Supplementary file 2 — Supplementary file2 (PNG 12 KB) Inquiring specialist of the nuclear scan [file 10554_2024_3085_MOESM2_ESM.png]
