# Supplementary material for: Detection of cardiac amyloidosis on routine bone scintigraphy: an important gatekeeper role for the nuclear medicine physician
Source: Int J Cardiovasc Imaging. 2024 Mar 23;40(6):1183–92. doi: 10.1007/s10554-024-03085-z (PMC11213735; doi:10.1007/s10554-024-03085-z)
Supplement: Supplementary file 3 — Supplementary file3 (DOCX 17 KB) [file 10554_2024_3085_MOESM3_ESM.docx]

|  |  | Missed | Diagnosed | Total |
| --- | --- | --- | --- | --- |
|  | | (n=38) | (n=2) | (n=40) |
| **hsTnT Tested** | | 6 (15.8%) | 0 | 6 (15.0%) |
|  | Number of Patients with an Elevated hsTnT | 6 (100%) | - | 6 (100%) |
|  | Median hsTnT (ng/L) *(IQR)* | 42.0  *(27.3 – 75)* | - | 42.0  *(27.3 – 75)* |
|  | Median Time from first Elevated hsTnT to Scan (days) *(IQR)* | 378  *(142 – 1141)* | - | 378  *(142 – 1141)* |
|  |  |  |  |  |
| **NT-proBNP Tested** | | 11 (28.9%) | 0 | 11 (27.5%) |
|  | Number of Patients with an Elevated NT-proBNP | 10 (90.9%) | - | 10 (90.9%) |
|  | Median Value (pg/mL) *(IQR)* | 98.0  *(41.1 – 138.5)* | - | 98.0  *(41.1 – 138.5)* |
|  | Median Time from first Elevated NT-proBNP to Scan (days) *(IQR)* | 1336  (748 – 1815) | - | 1336  (748 – 1815) |
|  | | | | |

Supplement table 1a: Laboratory Values (hsTnT and NT-proBNP) before a Nuclear Scan.

|  |  | Missed | Diagnosed | Total |
| --- | --- | --- | --- | --- |
|  | | (n=38) | (n=2) | (n=40) |
| **hsTnT Tested** | | 10 (26.3%) | 2 (100%) | 12 (30.0%) |
|  | Number of Patients with an Elevated hsTnT | 9 (90.0%) | 2 (100%) | 11 (91.7%) |
|  | Median hsTnT (ng/L) *(IQR)* | 30.5  *(19.5 – 45.8)* | 73.5 | 36.5  *(21.3 – 67.5)* |
|  | Median Time from Scan to first Elevated hsTnT (days) *(IQR)* | 420  *(230 – 607)* | 887 | 420  *(241 – 711)* |
|  |  |  |  |  |
| **NT-proBNP Tested** | | 14 (13.2%) | 2 (100%) | 16 (40%) |
|  | Number of Patients with an Elevated NT-proBNP | 14 (100%) | 2 (100%) | 16 (100%) |
|  | Median NT-proBNP (pg/mL) *(IQR)* | 193.5  *(59.5 – 411.8)* | 318.5 | 196.5  *(89.6 – 435.3)* |
|  | Median Time from Scan to first Elevated NT-proBNP (days) *(IQR)* | 350  *(141-1191)* | 902 | 350  *(186 – 1222)* |
|  | | | | |

Supplement table 1b: Laboratory Values (hsTnT and NT-proBNP) after a Nuclear Scan
