# Supplementary material for: Detection of cardiac amyloidosis on routine bone scintigraphy: an important gatekeeper role for the nuclear medicine physician
Source: Int J Cardiovasc Imaging. 2024 Mar 23;40(6):1183–92. doi: 10.1007/s10554-024-03085-z (PMC11213735; doi:10.1007/s10554-024-03085-z)
Supplement: Supplementary file 4 — Supplementary file4 (DOCX 19 KB) [file 10554_2024_3085_MOESM4_ESM.docx]

|  |  | Missed | Diagnosed | Total |
| --- | --- | --- | --- | --- |
|  | | (n=16) | (n=2) | (n=18) |
| **Dilated Left Atrium** | | 14 (88%) | 2 (100%) | 16 (89%) |
| **Left Ventricular Hypertrophy** | | 7 (44%) | 0 | 7 (39%) |
| **Preserved LVEF (>50%)** | | 12 (75%) | 1 (50%) | 13 (72%) |
| **Diastolic Dysfunction Grade ≥ 2** | | 3 (19%) | 1 (50%) | 4 (22%) |
| **Pericardial Fluid** | | 0 (0%) | 0 | 0 (0%) |
| **Wall Movement Disturbances** | | 4 (25%) | 0 | 4 (22%) |
| **Rhythm Disturbances** | |  |  |  |
|  | Atrial Fibrillation | 5 (32%) | 0 | 5 (27%) |
|  | Ventricular Pacing | 1 (6%) | 0 | 1 (6%) |
|  |  |  |  |  |

Supplement table 2a: Echocardiography Abnormalities prior to a positive nuclear scan.

|  |  | Valid, | Missed | Valid, | Diagnosed | Valid, | Total |
| --- | --- | --- | --- | --- | --- | --- | --- |
|  |  | n | (n=16) | n | (N=2) | n | (N=18) |
| **Body Surface Area** (m^2^) | | 16 | 1.97 [1.90-2.11] | 2 | 1.98 | 18 | 1.97 [1.90-2.07] |
| **Left Atrium Function** | |  |  |  |  |  |  |
|  | LA Volume Index (mL/m^2^) | 16 | 41.65 [36.59-50.94] | 2 | 47.39 | 18 | 41.65 [36.89-51.62] |
| **Left Ventricular Function** | |  |  |  |  |  |  |
|  | LV Mass Index (g/m^2^) | 18 | 87.17 [67.58-100.65] | 2 | 81.24 | 18 | 82.79 [67.75-100.40] |
|  | IVS (mm) | 18 | 10 [9-11] | 2 | 9 | 18 | 10 [9-11] |
|  | LVPW (mm) | 18 | 10 [9-10] | 2 | 10 | 18 | 10 [9-10] |
|  | LVEDD (mm) | 18 | 50.5 [45.25-52.75] | 2 | 49.50 | 18 | 50.50 [45.75-52.25] |
|  | LVEF Teichholz (%) | 18 | 55 [48-58] | 2 | 50 | 18 | 55 [47-59] |
| **Diastolic Function** | |  |  |  |  |  |  |
|  | E/A | 10 | 0.79 [0.69-0.93] | 1 | 0.77 | 12 | 0.79 [0.67-0.95] |
|  | E/e' IVS | 3 | 12.76 [9.46-12-76] | 1 | 15.14 | 4 | 13.41 [10.28-14.87] |
|  | TI Velocity (m/sec) | 15 | 2.30 [2.20-2.70] | 1 | 2.80 | 16 | 2.30 [2.23-2.70] |
| **Time to First Abnormal Echo** (days) | | 16 | 1313 [860-2036] | 2 | 1386 | 18 | 1313 [941-1992] |
|  | |  |  |  |  |  |  |

Supplement table 2b: Echocardiography Abnormalities prior to a positive nuclear scan.

Data presented as n (%) or median [interquartile range].

Abbreviations: LA = Left Atrium, LV = Left Ventricle, IVS = Interventricular septum thickness, LVPW = Left Ventricle Posterior Wall thickness, LVEF =Left Ventricular Ejection Fraction, AVA = Aortic Valve Area, E/A = peak early mitral inflow velocity/peak late mitral inflow velocity, E/e’ = peak early mitral inflow velocity/ peak early diastolic mitral annular velocity, TI = tricuspid valve insufficiency
