# Supplementary material for: Detection of cardiac amyloidosis on routine bone scintigraphy: an important gatekeeper role for the nuclear medicine physician
Source: Int J Cardiovasc Imaging. 2024 Mar 23;40(6):1183–92. doi: 10.1007/s10554-024-03085-z (PMC11213735; doi:10.1007/s10554-024-03085-z)
Supplement: Supplementary file 5 — Supplementary file5 (DOCX 19 KB) [file 10554_2024_3085_MOESM5_ESM.docx]

|  |  | Missed | Diagnosed | Total |
| --- | --- | --- | --- | --- |
|  | | (n=17) | (n=2) | (n=19) |
| **Dilated Left Atrium** | | 13 (76%) | 2 (100%) | 15 (79%) |
| **Left Ventricular Hypertrophy** | | 7 (41%) | 2 (100%) | 9 (47%) |
| **Preserved LVEF (>50%)** | | 9 (53%) | 1 (50%) | 10 (53%) |
| **Diastolic Dysfunction Grade ≥ 2** | | 3 (18%) | 1 (50%) | 4 (21%) |
| **Pericardial Fluid** | | 0 | 0 | 0 |
| **Wall Movement Disturbances** | | 5 (29%) | 1 (50%) | 6 (32%) |
| **Rhythm Disturbances** | |  |  |  |
|  | Atrial Fibrillation | 7 (41%) | 0 | 7 (37%) |
|  | Ventricular Pacing | 3 (18%) | 1 (50%) | 4 (21%) |
|  |  |  |  |  |

Supplement table 3a: Echocardiography Abnormalities at any time after a positive nuclear scan.

|  |  |  | Missed |  | Diagnosed |  | Total |
| --- | --- | --- | --- | --- | --- | --- | --- |
|  | | Valid,  n | (n=17) | Valid,  n | (n=2) | Valid,  n | (n=19) |
| **Body Surface Area** (m^2^) | | 17 | 1.94 [1.88-2.08] | 2 | 1.97 | 19 | 1.94 [1.88-2.08] |
| **Left Atrium Function** | |  |  |  |  |  |  |
|  | LA Volume Index (mL/m^2^) | 17 | 37.55 [23.70-59.91] | 2 | 49.60 | 19 | 38.52 [25.94-55.31] |
| **Left Ventricular Function** | |  |  |  |  |  |  |
|  | LV Mass Index (g/m^2^) | 17 | 73.56 [60.23-91.15] | 2 | 118.26 | 19 | 79.77 [61.02-106.23] |
|  | IVS (mm) | 17 | 9 [9-11] | 2 | 13 | 19 | 9 [9-12] |
|  | LVPW (mm) | 17 | 9 [9-10] | 2 | 12 | 19 | 9 [9-10] |
|  | LVEDD (mm) | 17 | 48.00 [44.00-51.00] | 2 | 49.00 | 19 | 48.00 [44.00-52.00] |
|  | LVEF Teichholz (%) | 17 | 57 [42-64] | 2 | 59 | 19 | 57 [42-62] |
| **Diastolic Function** | |  |  |  |  |  |  |
|  | E/A | 11 | 0.92 [0.74-1.09] | 1 | 0.87 | 12 | 0.91 [0.77-1.08] |
|  | E/e' IVS | 11 | 10.82 [9.06-13.17] | 1 | 32.19 | 12 | 11.06 [9.17-13.88] |
|  | TI Velocity (m/sec) | 15 | 2.50 [2.30-2.70] | 2 | 2.70 | 17 | 2.50 [2.30-2.70] |
| **Time to First Abnormal Echo** (days) | | 17 | 301 [137-525] | 2 | 696 | 19 | 301 [149-538] |
|  | |  |  |  |  |  |  |

Supplement table 3b: Echocardiography first available evaluation after a positive nuclear scan.

Data presented as n (%) or median [interquartile range].

Abbreviations: LA = Left Atrium, LV = Left Ventricle, IVS = Interventricular septum thickness, LVPW = Left Ventricle Posterior Wall thickness, LVEF =Left Ventricular Ejection Fraction, AVA = Aortic Valve Area, E/A = peak early mitral inflow velocity/peak late mitral inflow velocity, E/e’ = peak early mitral inflow velocity/ peak early diastolic mitral annular velocity, TI = tricuspid valve insufficiency
