# Supplementary material for: Detection of cardiac amyloidosis on routine bone scintigraphy: an important gatekeeper role for the nuclear medicine physician
Source: Int J Cardiovasc Imaging. 2024 Mar 23;40(6):1183–92. doi: 10.1007/s10554-024-03085-z (PMC11213735; doi:10.1007/s10554-024-03085-z)
Supplement: Supplementary file 6 — Supplementary file6 (DOCX 21 KB) [file 10554_2024_3085_MOESM6_ESM.docx]

|  |  | Missed | Diagnosed | Total |
| --- | --- | --- | --- | --- |
|  | | (n=33) | (n=2) | (n=35) |
| Sinus rhythm | | 28 (84.3%) | 2 (100.0%) | 30 (85.8%) |
| Heart frequency (beats/min) | | 73 [66-88] | 65 | 73 [64-85] |
| PQ duration (ms) | | 177 [161-207] | 371 | 179 [163-223] |
| QRS duration (ms) | | 98 [81-109] | 140 | 98 [82-120] |
| **Abnormal ECG** | | 22 (66.7%) | 2 (100.0%) | 24 (68.6%) |
| Abnormal heart axis | | 6 (18.2%) | 2 (100.0%) | 8 (22.9%) |
| Micro voltage | | 5 (15.2%) | 0 (0.0%) | 5 (14.9%) |
| Left ventricular hypertrophy | | 1 (3.0%) | 0 (0.0%) | 1 (2.9%) |
| Delayed intraventricular conduction (QRS ≥100ms) | | 14 (42.4%) | 2 (100.0%) | 16 (45.7%) |
| Atrioventricular block  First degree  Second degree  Third degree | | 4 (12.1%)  3 (9.1%)  1 (3.0%)  0 (0.0%) | 2 (100.0%)  2 (100.0%)  0 (0.0%)  0 (0.0%) | 6 (17.1%)  5 (14.3%)  1 (2.9%)  0 (0.0%) |
| Pseudo infarct pattern | | 5 (15.2%) | 1 (50.0%) | 6 (17.1%) |
| Delayed R wave propagation | | 9 (27.3%) | 0 (0.0%) | 9 (25.7%) |
| Abnormal repolarization | | 6 (18.2%) | 0 (0.0%) | 6 (17.1%) |
|  |  |  |  |  |

Supplement table 4a: Electrocardiography abnormalities prior to a positive nuclear scan.
Data presented as n (%) or median [interquartile range].

|  |  | Missed | Diagnosed | Total |
| --- | --- | --- | --- | --- |
|  | | (n=32) | (n=2) | (n=34) |
| Sinus rhythm | | 22 (68.8%) | 1 (50.0%) | 23 (67.6%) |
| Heart frequency (beats/min) | | 76 [66-86] | 75 | 76 [65-88] |
| PQ duration (ms) | | 176 [164-192] | 250 | 176 [165-198] |
| QRS duration (ms) | | 94 [82-115] | 144 | 97 [83-116] |
| **Abnormal ECG** | | 28 (87.5%) | 2 (100.0%) | 30 (88.2%) |
| Abnormal heart axis | | 7 (21.9%) | 1 (50.0%) | 8 (23.5%) |
| Micro voltage | | 6 (18.8%) | 0 (0.0%) | 6 (17.6%) |
| Left ventricular hypertrophy | | 3 (9.4%) | 0 (0.0%) | 3 (8.8%) |
| Delayed intraventricular conduction (QRS ≥100ms) | | 15 (46.9%) | 1 (50.0%) | 16 (47.1%) |
| Atrioventricular block  First degree  Second degree  Third degree | | 5 (15.6%)  5 (15.6%)  0 (0.0%)  0 (0.0%) | 1 (50.0%)  1 (50.0%)  0 (0.0%)  0 (0.0%) | 6 (17.6%)  6 (17.6%)  0 (0.0%  0 (0.0%) |
| Pseudo infarct pattern | | 8 (25.0%) | 1 (50.0%) | 9 (26.5%) |
| Delayed R wave propagation | | 15 (46.9%) | 0 (0.0%) | 15 (44.1%) |
| Abnormal repolarization | | 8 (25.0%) | 1 (50.0%) | 9 (26.5%) |
|  |  |  |  |  |

Supplement table 4b: Electrocardiography abnormalities after positive nuclear scan.
Data presented as n (%) or median [interquartile range].
